# Supplementary material for: Uteroplacental Insufficiency Causes Microbiota Disruption and Lung Development Impairment in Growth-Restricted Newborn Rats
Source: Nutrients. 2022 Oct 19;14(20):4388. doi: 10.3390/nu14204388 (PMC9608653; doi:10.3390/nu14204388)

Figure S1: Heatmap of intestinal microbiota in mothers.

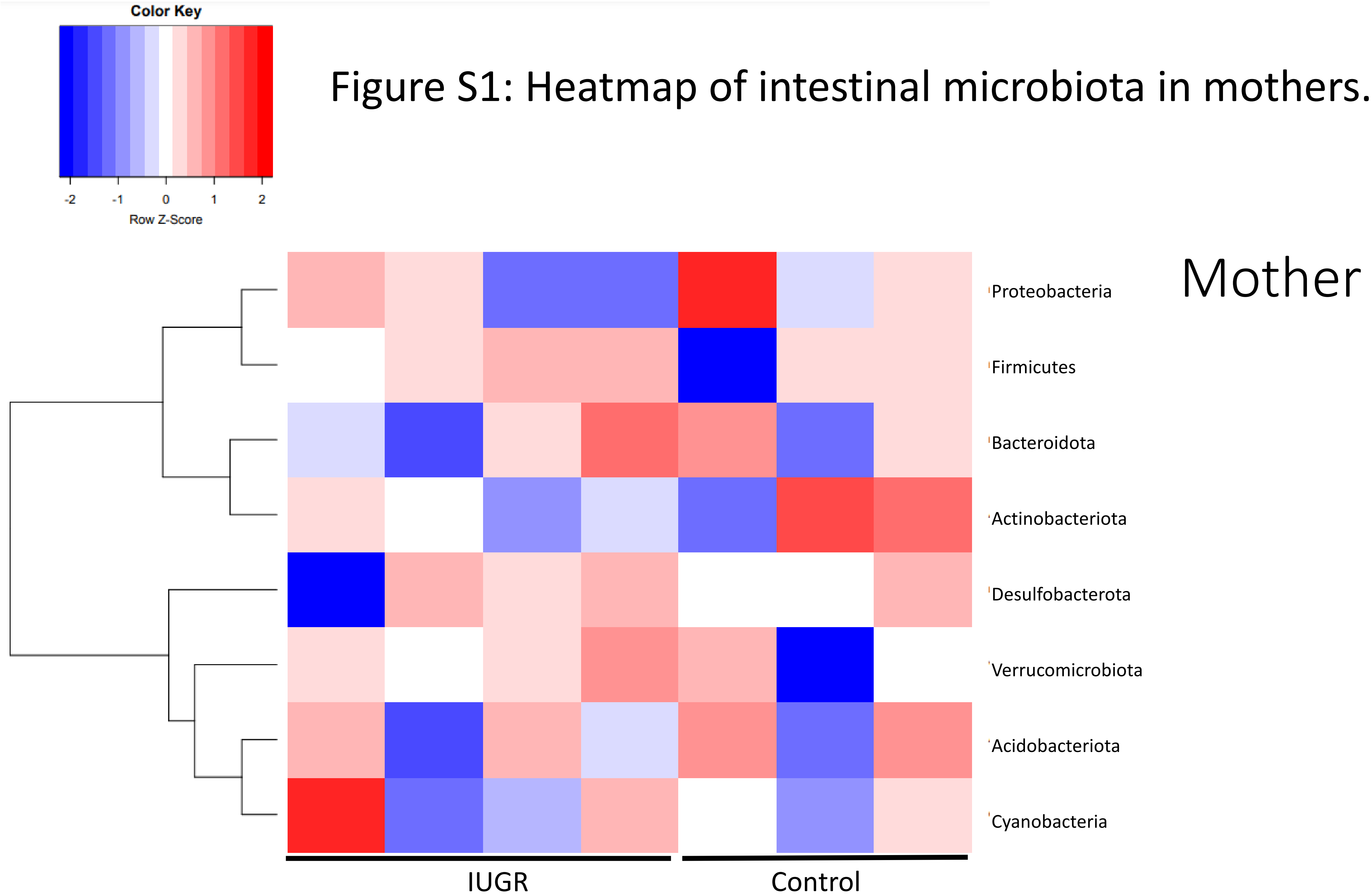

Figure S2: Heatmap of lung microbiota in rats on postnatal day 0.

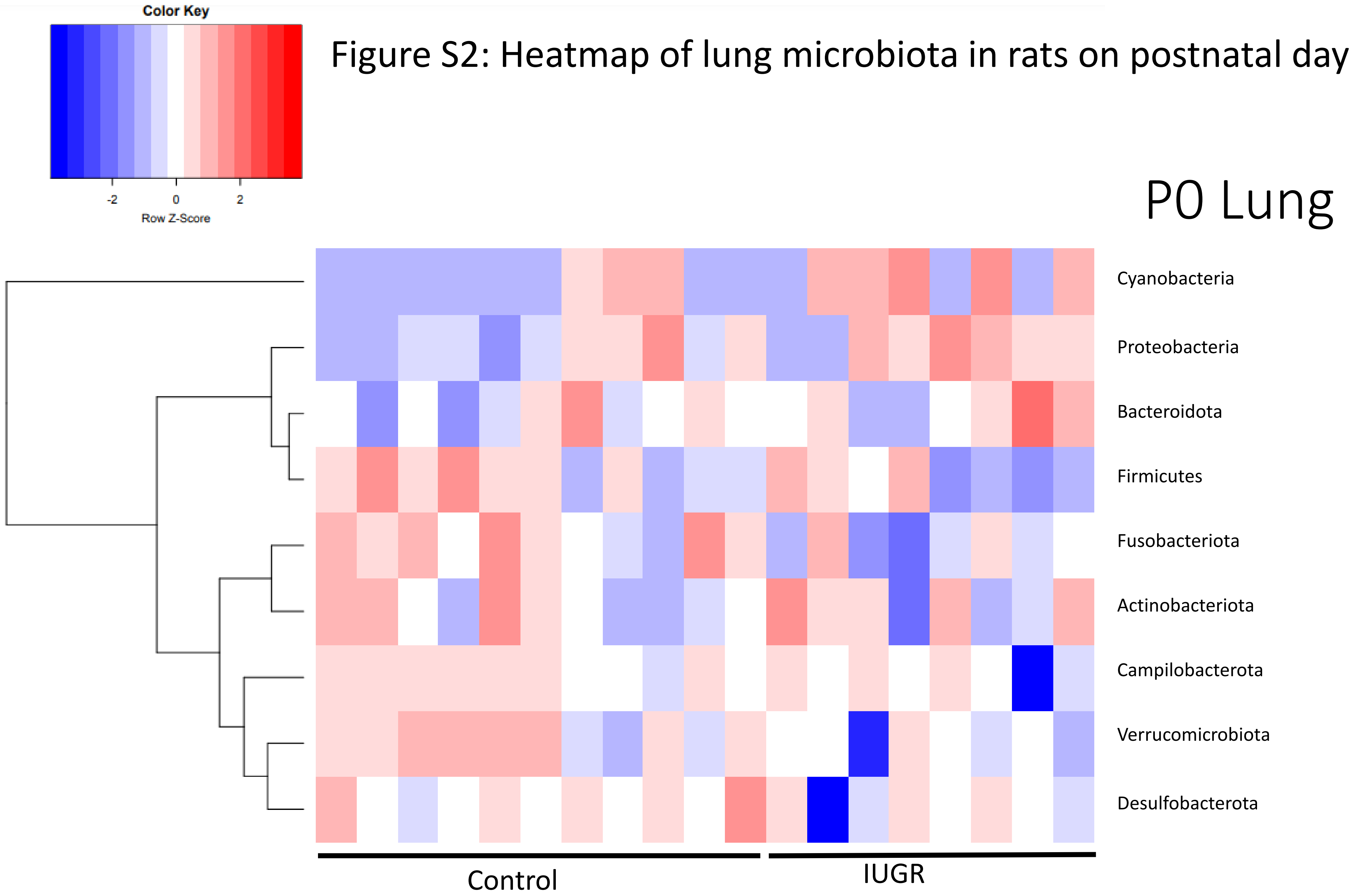

Figure S3: Heatmap of intestinal microbiota in rats on postnatal day 0.

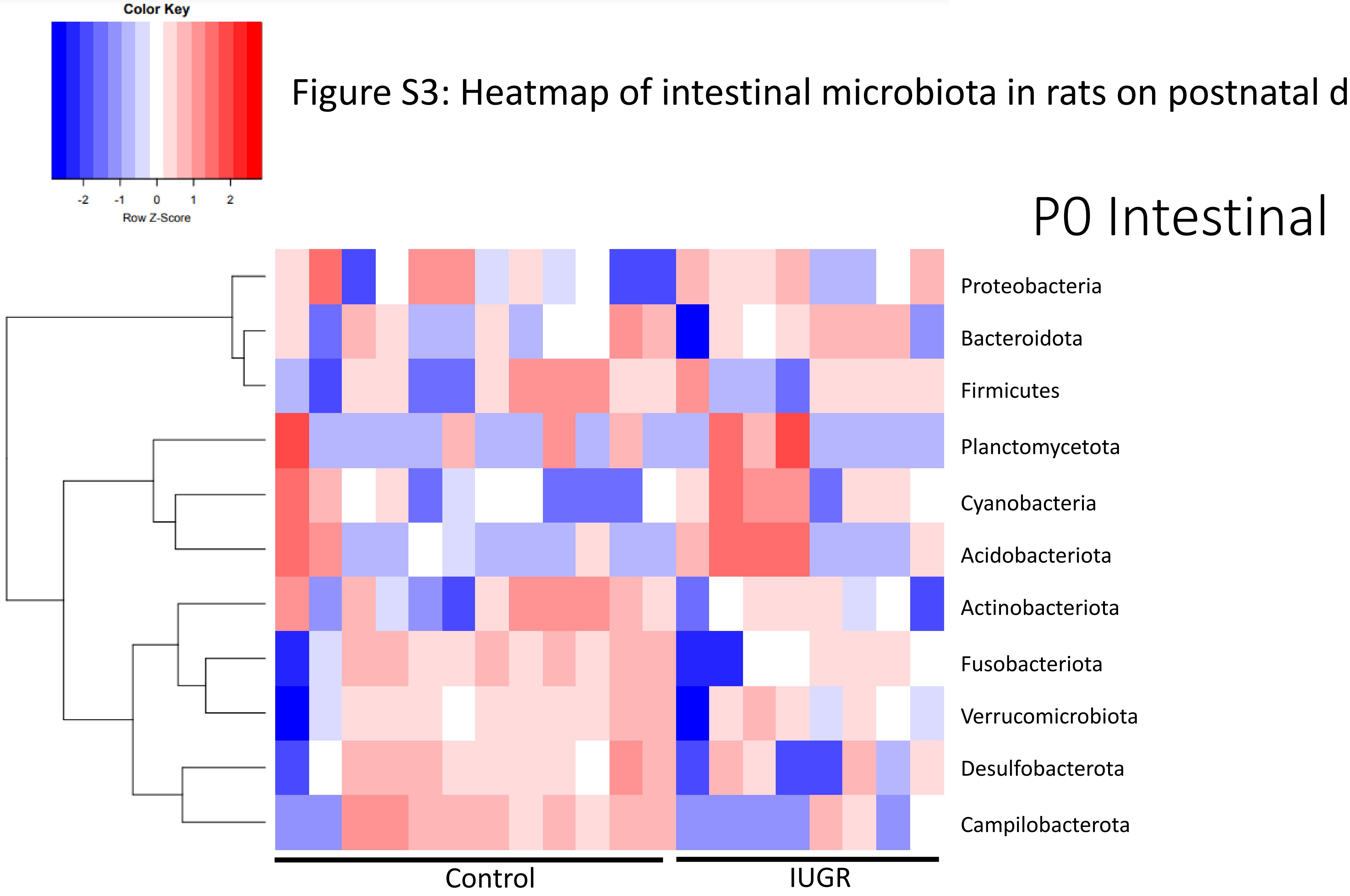

Figure S4: Heatmap of lung microbiota in rats on postnatal day 7.

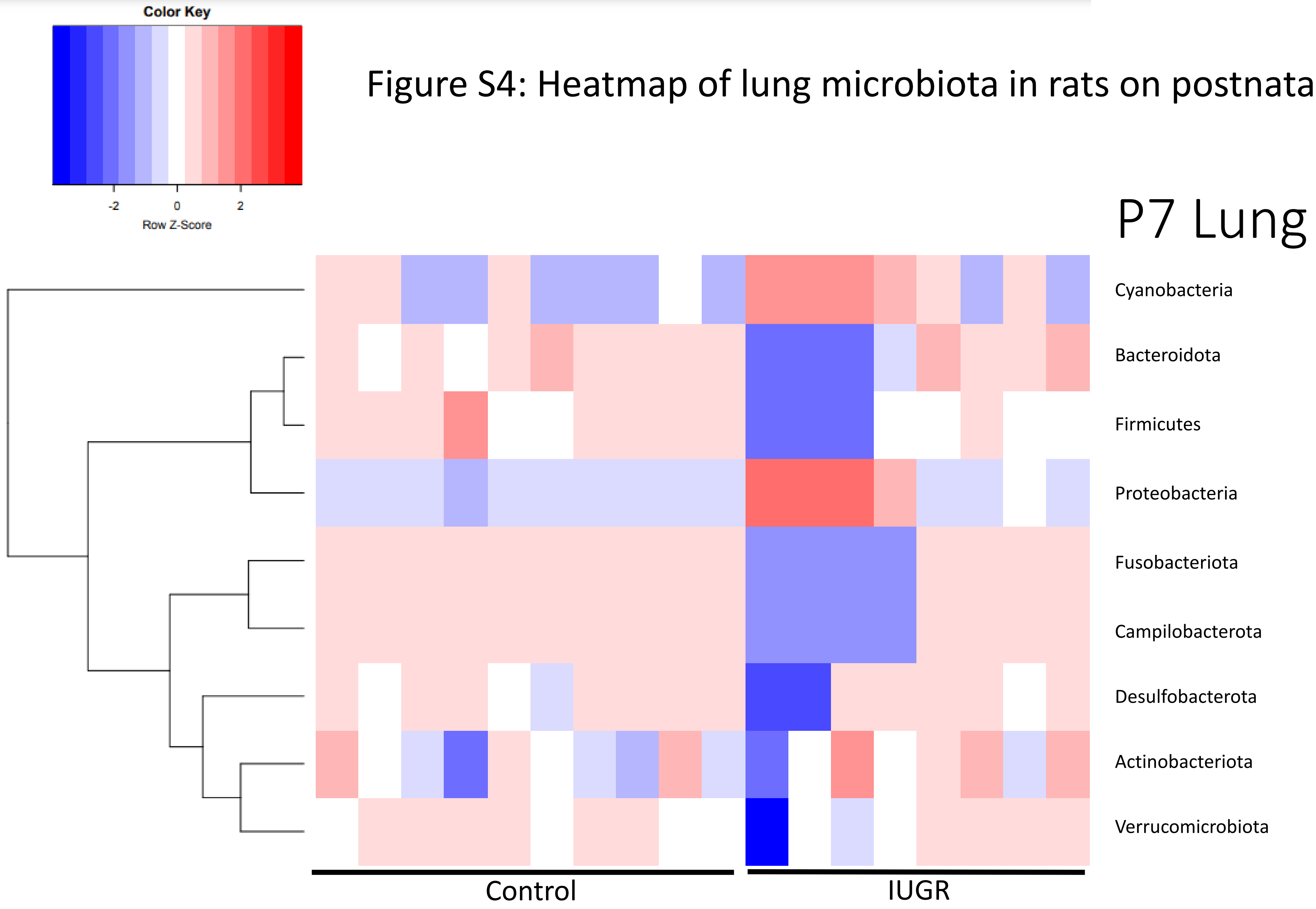

Figure S5: Heatmap of intestinal microbiota in rats on postnatal day 7.

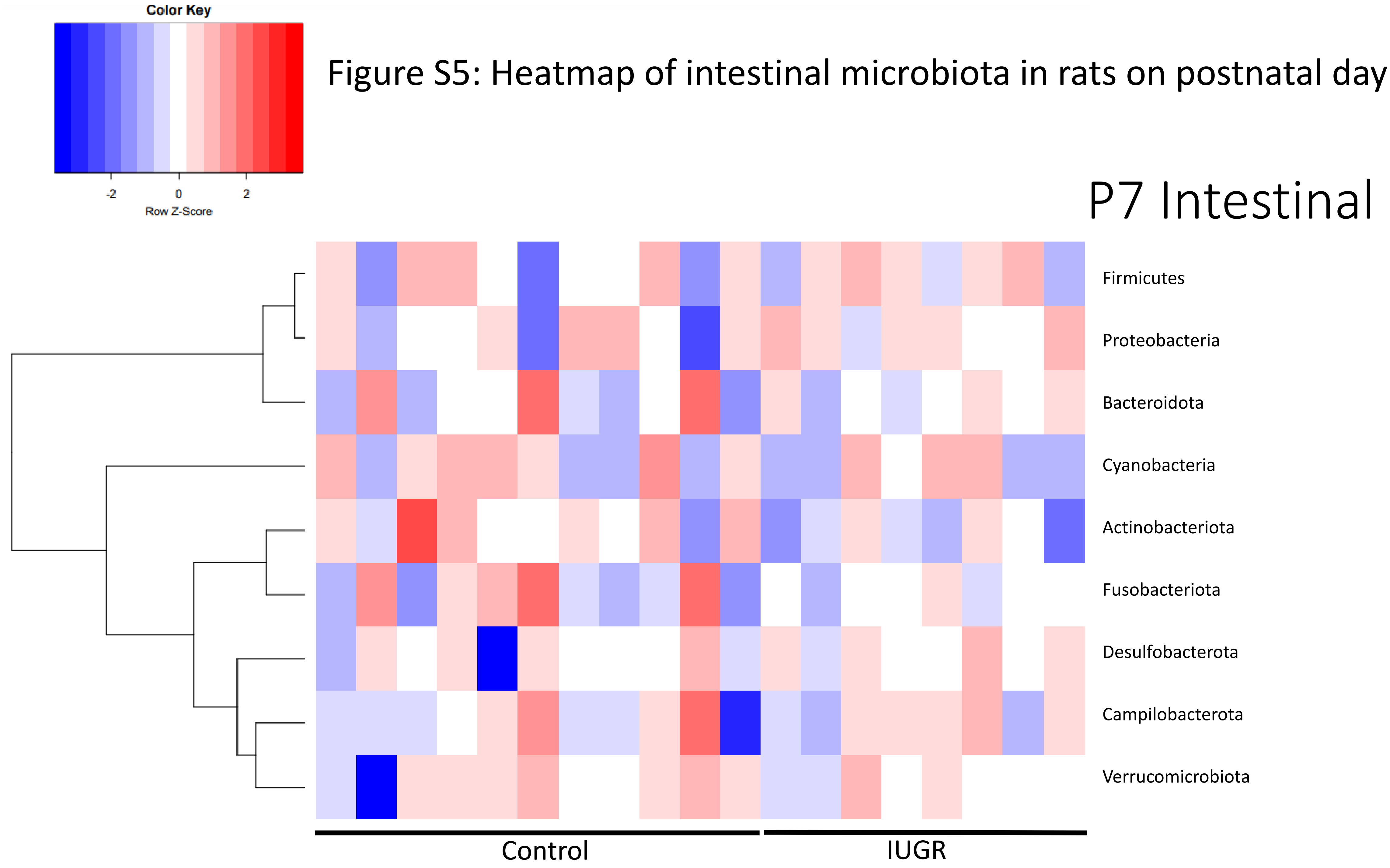

Supplement: Supplementary file 1 [file nutrients-14-04388-s001.zip › nutrients-1958934-supplementary.pdf]
